# Supplementary material for: Introduction and adoption of innovative invasive procedures and devices in the NHS: an in-depth analysis of written policies and qualitative interviews (the INTRODUCE study protocol)
Source: BMJ Open. 2019 Aug 26;9(8):e029963. doi: 10.1136/bmjopen-2019-029963 (PMC6719760; doi:10.1136/bmjopen-2019-029963)

Supplementary file 1. Figure showing the relationship between NICE and the NHS, including roles and structure

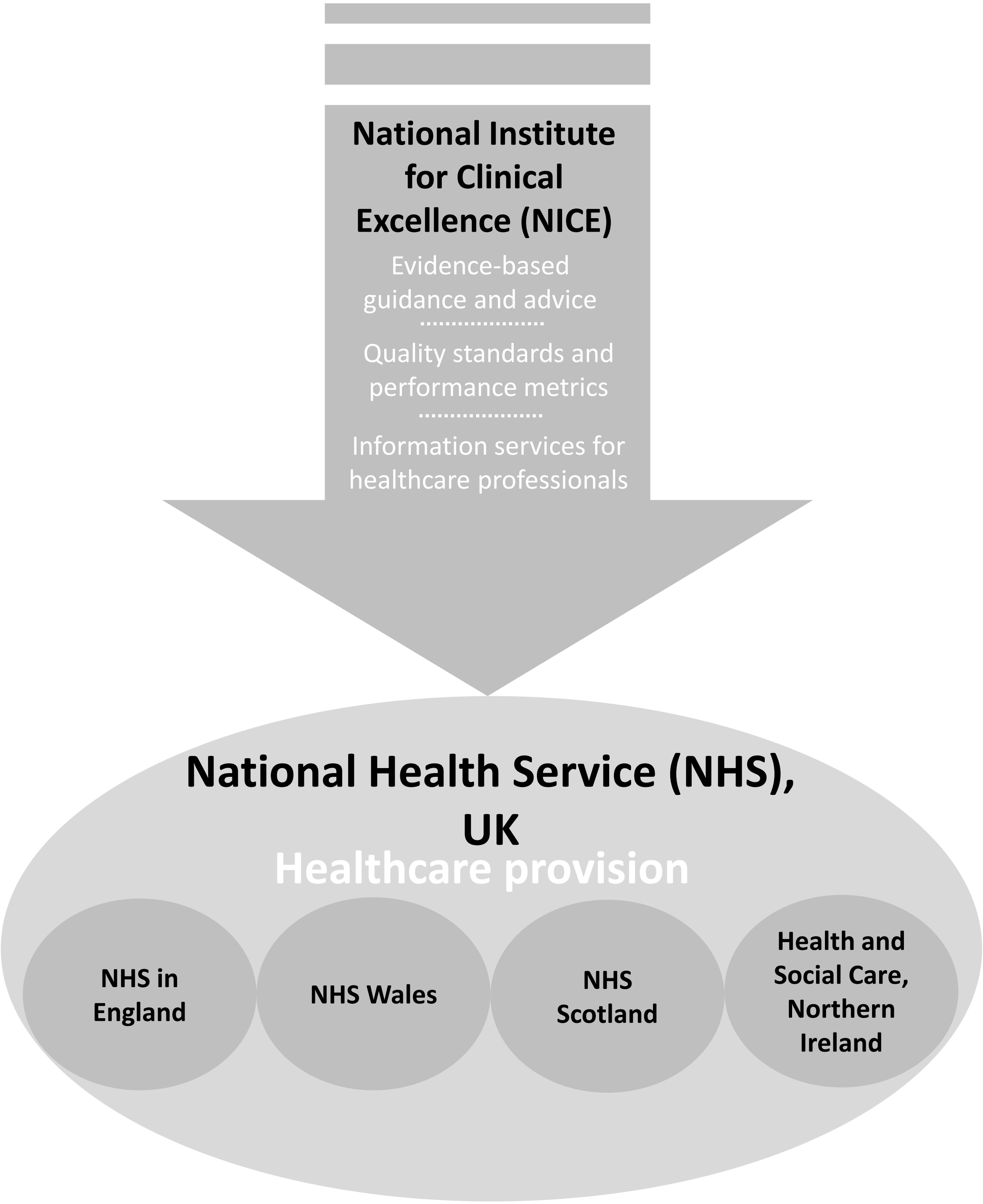

Supplement: Supplementary data [file bmjopen-2019-029963supp001.pdf]
