# Supplementary material for: Introduction and adoption of innovative invasive procedures and devices in the NHS: an in-depth analysis of written policies and qualitative interviews (the INTRODUCE study protocol)
Source: BMJ Open. 2019 Aug 26;9(8):e029963. doi: 10.1136/bmjopen-2019-029963 (PMC6719760; doi:10.1136/bmjopen-2019-029963)
Supplement: Supplementary data [file bmjopen-2019-029963supp002.pdf]

Supplementary file 2. Preliminary data extraction form

**DATA EXTRACTION DETAILS**			
**1**	**Who is extracting the data? (initials)**		
	[ ][ ][ ]		
**2**	**What is the name of the trust?**		
**3**	**What is the Trust ID?**		
	[ ][ ][ ]		
**4**	**Policy title:**		
**5**	**Current policy and/or version number:**		
	[ ][ ][ ]		
**SCOPE OF POLICY**			
**1**	**What procedures/devices does the policy apply to?**		
**a)**	**Invasive procedures**		
	☐ Yes	☐ No	☐ N/A
	If yes, answer i) - ii). If no or not stated go to question b).		
	**i) What term is used to describe these procedures?**		
	**ii) Does the policy define invasive procedures (or similar term used)?**		
	☐ Yes	☐ No	
	If yes -		
	• Copy and paste definition:		
**b)**	**Invasive devices**		
	☐ Yes	☐ No	☐ N/A
	If yes answer i)-iv). If no or not stated go to c).		
	**i) What term is used to describe invasive devices?**		
	**ii) Does the policy define invasive devices (or similar term used)?**		
	☐ Yes	☐ No	
	If yes -		
	• Copy and paste definition:		

**2**	**When should the policy be implemented?**		
**a)**	**When procedures/devices are new (or a similar term that implies this)?**		
	☐ Yes (explicitly states that the policy should be implemented in this instance)		
	☐ No (explicitly states that the policy should not be implemented in this instance)		
	☐ Unclear		
	☐ Not stated (does not state whether or not policy should be implemented in this instance)		
	If yes, no or unclear answer i)-iii). If not stated go to b).		
	**i) Does the policy define 'new' (or similar term)?**		
	☐ Yes	☐ No	
**b)**	**When procedures/devices are modified (to include references to 'changes in clinical practice') (or a similar term that implies this)?**		
	☐ Yes (explicitly states that the policy should be implemented in this instance)		
	☐ No (explicitly states that the policy should not be implemented in this instance)		
	☐ Unclear		
	☐ Not stated (does not state whether or not policy should be implemented in this instance)		
	If yes, no or unclear answer i)-iii). If not stated go to c).		
	**i) Does the policy define 'modification' (or similar term)?**		
	☐ Yes	☐ No	
**c)**	**When a procedure/device is used within a research study**		
	☐ Yes (explicitly states that the policy should be implemented in this instance)		
	☐ No (explicitly states that the policy should not be implemented in this instance)		
	☐ Unclear		
	☐ Not stated (does not state whether or not policy should be implemented in this instance)		
	i) Copy and paste all text: (if none, type N/A) [QUALITATIVE DATA]		
**d)**	**When procedures/devices approved by research ethics are used outside of the specified protocol**		
	☐ Yes (explicitly states that the policy should be implemented in this instance)		
	☐ No (explicitly states that the policy should not be implemented in this instance)		

	☐ Unclear		
	☐ Not stated (does not state whether or not policy should be implemented in this instance)		
	i) Copy and paste all text: (if none, type N/A) [QUALITATIVE DATA]		
**e)**	**Other. Please state any other instances in which the policy should be implemented (if none, type N/A):**		
**PATIENT INFORMATION AND CONSENT**			
**1**	**Does the policy state that patients should receive information and/or complete a consent form that is specific to the new procedure/device delivered?**		
	☐ Yes	☐ No	☐ Unclear
	If yes or unclear answer a)-c). If no go to section I.		
**a)**	**Is the applicant required to submit the patient information leaflet and/or consent form with the application?**		
	☐ Yes	☐ No	☐ Unclear
**b)**	**Does the policy provide guidance on what details should be included in the patient information leaflet and/or consent form?**		
	☐ Yes	☐ No	☐ Unclear
	If yes, answer i). If no or unclear go to section I.		
	**i) Does this include informing patients that the procedure/device is new/modified?**		
	☐ Yes	☐ No	☐ Unclear
**c)**	**Does the policy state that specific PIL information should be provided for all approved procedures/devices?**		
	Yes - specific PIL information should be provided for all new procedures/devices.		
	No - specific PIL information is only required in a subset of new procedures/devices (i.e. if there is no NICE Guidance in place).		
	Not stated		
**AUDIT AND OUTCOME MONITORING**			
**1**	**Does the policy state that the use of new procedures/devices should be monitored/reviewed in any way?**		
	☐ Yes	☐ No	☐ N/A
	If yes, go to question 2. If no or not stated go to section H.		
**2**	**Does the policy require applicants to submit outcome data, including adverse events, clinical complications etc?**		

	☐ Yes	☐ No	☐ Unclear
	If yes, answer a)-d). If no or unclear go to question 4.		
a)	**Who is responsible for submitting monitoring/outcome data? (tick all that apply)**		
	☐ Individual clinician		
	☐ Head of department		
	☐ Other. Please state:		
	☐ Not stated		
b)	**Who is the monitoring/outcome data submitted too? (tick all that apply)**		
	☐ Trust committee responsible for the introduction of new procedures/devices		
	☐ NICE		
	☐ Audit & Effectiveness Dept/Group		
	☐ Clinical effectiveness/safety group		
	☐ Clinical audit group		
	☐ Medical and/or Nursing Director		
	☐ Clinical governance group		
	☐ Other. Please state:		
	☐ Not stated		
c)	**Does the policy specify the type of monitoring/outcome data to be submitted?**		
	☐ Yes	☐ No	
	If yes, answer i)-ii). If no go to question d).		
	i) Which of the following outcomes are specified? (tick all that apply)		
	☐ Adverse events		
	☐ Safety		
	☐ Effectiveness		
	☐ Cost related outcomes		
	☐ Near misses		
	☐ Patient Experience/PROMS		
	☐ Other. Please state:		
3	**When does the policy state that the procedure should be reviewed/outcome data submitted?**		
	☐ After a certain length of time. *If selected answer a).*		
	☐ After a certain number of cases. *If selected answer b).*		
	☐ This is decided on a case by case basis or according to level of approval/recommendation given by the committee/group.		
	☐ Unclear. *If selected answer c)*		
a)	**How many months?**		
	[ ][ ][ ][ ]		
	Go to question 6		
b)	**How many cases?**		

	[ ][ ][ ][ ]		
	Go to question 6		
**c)**	**Copy and paste text:**		
**4**	**Does the policy outline when the procedure/device may be used in clinical practice without continuing review via this policy? (ie. when the procedure/device can be adopted in to routine clinical practice)**		
	☐ Yes	☐ No	☐ Unclear
	If yes or unclear, answer a). If no go to question 8.		
**a)**	**Copy and paste text:**		
**5**	**Does the policy outline when or why the use of a new procedure may be stopped/abandoned/immediately reported to MD?**		
	☐ Yes	☐ No	☐ Unclear
	If yes or unclear, answer a). If no go to section H.		
**a)**	**Copy and paste text:**		
**ADDITIONAL NOTES**			
**1**	**Please record any additional comments here. These may relate to data extraction or may be reflections on the policy.**		
