# Supplementary material for: Introduction and adoption of innovative invasive procedures and devices in the NHS: an in-depth analysis of written policies and qualitative interviews (the INTRODUCE study protocol)
Source: BMJ Open. 2019 Aug 26;9(8):e029963. doi: 10.1136/bmjopen-2019-029963 (PMC6719760; doi:10.1136/bmjopen-2019-029963)
Supplement: Supplementary data [file bmjopen-2019-029963supp003.pdf]

Supplementary file 3. Example preliminary topic guide for interviews with clinicians

- Introductory remarks, including taking verbal consent

- Can you tell me about yourself?
    - What is your current position and role?
    - Can you describe the range /type(s) of surgical procedures you undertake?
    - Particular areas of interest/expertise?

- As you know, we are looking at innovation in surgery. Can you talk me through how you would define innovation? (Newness; degree of change, level of risk, impact?)

- Have you experienced an innovative procedure being implemented into practice?
    - If so, can you describe it to me? What made it innovative?
    - What about a time when you have had to learn a new procedure?
    - Potential Prompts: How/where implemented? How did you learn about it? Any changes/refinements over time?
    - Was the procedure monitored in anyway?
    - What has happened to it now?

- If you or a colleague did develop an innovative procedure, do you think ethics approval is needed?
- Is there a procedure for implementing an innovative procedure at your hospital?
- Do you think patients should be informed about the new procedure? What aspects important to communicate?
    - How do you think patients will respond to innovative procedures? What does ‘informed consent’ mean to you?

- Ending the interview: Is there anything else that I haven’t mentioned? Anyone else you recommend I could contact?
