# Supplementary material for: Introduction and adoption of innovative invasive procedures and devices in the NHS: an in-depth analysis of written policies and qualitative interviews (the INTRODUCE study protocol)
Source: BMJ Open. 2019 Aug 26;9(8):e029963. doi: 10.1136/bmjopen-2019-029963 (PMC6719760; doi:10.1136/bmjopen-2019-029963)
Supplement: Author's manuscript [file bmjopen-2019-029963.draft_revisions.pdf]

BMJ Open is committed to open peer review. As part of this commitment we make the peer review history of every article we publish publicly available.

When an article is published we post the peer reviewers' comments and the authors' responses online. We also post the versions of the paper that were used during peer review. These are the versions that the peer review comments apply to.

The versions of the paper that follow are the versions that were submitted during the peer review process. They are not the versions of record or the final published versions. They should not be cited or distributed as the published version of this manuscript.

BMJ Open is an open access journal and the full, final, typeset and author-corrected version of record of the manuscript is available on our site with no access controls, subscription charges or pay-per-view fees (http://bmjopen.bmj.com).

If you have any questions on BMJ Open's open peer review process please email info.bmjopen@bmj.com

BMJ Open

An investigation into NHS policy and practice for the introduction and adoption of innovative invasive procedures and devices: the INTRODUCE study protocol

Journal:	*BMJ Open*
Manuscript ID	bmjopen-2019-029963
Article Type:	Protocol
Date Submitted by the Author:	19-Feb-2019
Complete List of Authors:	Cousins, Sian; University of Bristol, Population Health Sciences<br>Richards, Hollie; University of Bristol, Population Health Sciences<br>Zahra, Jesmond; University of Bristol, Population Health Sciences<br>Elliott, Daisy; University of Bristol, Population Health Sciences<br>Avery, Kerry; University of Bristol, Population Health Sciences<br>Robertson, Harry; University of Bristol, Population Health Sciences<br>Paramasivan, Sangeetha ; University of Bristol, Population Health Sciences<br>Wilson, Nicholas; University of Bristol, Population Health Sciences<br>Mathews, Johnny; University of Bristol, Population Health Sciences<br>Tolkien, Zoe; University of Bristol, Population Health Sciences<br>Main, Barry; University of Bristol, Population Health Sciences; University Hospitals Bristol NHS Foundation Trust<br>Blencowe, Natalie; University of Bristol, Population Health Sciences; University Hospitals Bristol NHS Foundation Trust<br>Hinchliffe, Robert; University of Bristol, Population Health Sciences; North Bristol NHS Trust<br>Blazeby, Jane; University of Bristol, Population Health Sciences; University Hospitals Bristol NHS Foundation Trust
Keywords:	SURGERY, innovation, NHS, Clinical governance < HEALTH SERVICES ADMINISTRATION & MANAGEMENT

SCHOLARONE™
Manuscripts

An investigation into NHS policy and practice for the introduction and adoption of innovative invasive procedures and devices: the INTRODUCE study protocol

Cousins S,[1] Richards HS,[1] Zahra J,[1] Elliott D,[1] Avery KNL,[1] Robertson HF,[1] Paramasivan S,[1] Wilson N,[1] Mathews JA,[1] Tolkien Z,[1] Main BG,[1,2] Blencowe NS,[1,2] Hinchliffe R,[1,3] Blazeby JM[1,2]

[1]National Institute for Health Research Bristol Biomedical Research Centre Surgical Innovation Theme and the Medical Research Council ConDuCT-II (Collaboration and innovation in Difficult and Complex randomised controlled Trials In Invasive procedures) Hub for Trials Methodology Research, Centre for Surgical Research, Population Health Sciences, Bristol Medical School, University of Bristol, 39 Whatley Road, Clifton, Bristol, UK. BS8 2PS;

[2]University Hospitals Bristol NHS Foundation Trust, Bristol, UK. BS2 8HW

[3]North Bristol NHS Trust, Bristol, UK. BS10 5NB

Corresponding author: Sian Cousins, Sian.cousins@bristol.ac.uk

Word count: 3094 (4000 max)

For peer review only

Abstract

Introduction

Innovation is key to improving outcomes in healthcare. Innovative pharmaceutical products undergo rigorous phased research evaluation before they are introduced into practice. The introduction of innovative invasive procedures and devices is much less rigorous and phased research, including randomised controlled trials, is not always undertaken. Whilst the innovator (usually a surgeon) may introduce a new or modified procedure/device within the context of formal research, they may also be introduced by applying for local NHS organisation approval alone. Written policies for the introduction of new procedures and/or devices often form part of this local clinical governance infrastructure; however, little is known about their content or use in practice.

This study aims to systematically investigate how new invasive procedures and devices are introduced in NHS England and Wales.

Methods and analysis

An in-depth analysis of written policies will be undertaken. This will be supplemented with interviews with key stakeholders.

All acute NHS trusts in England and Health Boards in Wales will be systematically approached and asked to provide written policies for the introduction of new invasive procedures and devices. Information on the following will be captured: I) policy scope, including when new procedures should be introduced within a formal research framework; II) requirements for patient information provision; III) outcome reporting and/or monitoring. Data will be extracted using a standardised form developed iteratively within the study team.

Semi-structured interviews with medical directors, audit and governance leads, and surgeons will explore views regarding the introduction of new invasive procedures into practice, including knowledge of and implementation of current policies.

For peer review only - http://bmjopen.bmj.com/site/about/guidelines.xhtml

Ethics and dissemination

In-depth analysis of written policies does not require ethics approval. The University of Bristol Ethics Committee (56522) approved the interview component of the study. Findings from this work will be presented at appropriate conferences and will be published in peer-reviewed journals.

Article summary

Strengths and limitations of this study

- This is the first systematic study addressing how the NHS introduces innovative invasive procedures into clinical practice; a topic of international public, professional and political interest given the wealth of historic and recent examples of patient harm caused by lack of regulation.
- The study combines analysis of written policies and interviews with key stakeholders to ensure an in-depth exploration of the topic to inform national guidance to improve and standardise the introduction of innovative invasive procedures and devices.
- The degree to which policies are actually adhered to across local NHS organisations is not systematically explored in the current study and is recommended for future research.

Introduction

Invasive procedures are a fundamental part of healthcare and can include surgical operations with and without devices, as well as endoscopic and radiologically guided interventions. At least 230 million invasive procedures are delivered worldwide,[1] with 12.5 million undertaken in the United Kingdom (UK) annually.[2] This number is likely to increase with continued innovation, including the advent of new technologies and minimal access procedures.

Innovation in invasive procedures is key. It may include modifying existing techniques,[3] to performing completely new first-in-human invasive procedures.[4] Whilst innovation is common, the governance surrounding it is not standardised and is currently under much scrutiny.[5-7] A recent

For peer review only - http://bmjopen.bmj.com/site/about/guidelines.xhtml

For peer review only

inquest into the death of a patient following robot-assisted cardiac surgery, a procedure that had not been previously performed in the UK, found insufficient governance surrounding the introduction of this innovative procedure.[6] Recommendations from the Coroner, echoed by a statement from RCS,[8] included introducing stricter governance relating to the use of new technologies and procedures, specific measures to assess the competence and training received by clinicians wishing to undertake them, and detailed patient information provision regarding the risks associated with new procedures.

Innovation in invasive procedures may be introduced under the auspices of formal research studies, with a protocol and application for ethical approval. However, multiple reviews show that ethical approval is rarely gained when delivering innovative invasive procedures.[9-12] While medical devices to be used inside the body require a European Conformity (CE) mark[13-15] prior to use, the evidence required to gain this certification does not often come from high-quality randomised controlled trials and post-marketing surveillance is minimal.[7] This is very different from the tightly governed and transparent developmental pathways required for the introduction of new pharmaceutical procedures.[16]

Outside of research, innovative invasive procedures and devices may be introduced via local hospital policies. The UK National Institute for Clinical Excellence (NICE), which is responsible for improving health and social care in the NHS through evidence-based guidance, recommends that local NHS organisations (e.g. NHS trusts in England and health boards in Wales) have appropriate governance structures in place to review, authorise and monitor the introduction of new invasive procedures.[17] In addition, NICE recommends the approval of new invasive procedures that do not have existing NICE guidance should only be given if appropriate training of those delivering the procedure is demonstrated, patients are made aware of the new status of the procedures, and there are proposed arrangements for clinical audit.[18] These recommendations are echoed by organisations worldwide.[19-21] In the UK, it is the responsibility of local hospital organisations to implement this

For peer review only - http://bmjopen.bmj.com/site/about/guidelines.xhtml

guidance, although the extent to which this occurs is unknown, and there are instances where innovative procedures are introduced into practice without any formal governance.[6] Furthermore, where local policies do exist, little is known about their content or how they are used in practice; how trusts define a new procedure (i.e. when the guidance should be applied), what information should be given to patients, and how outcomes of new procedures are recorded and monitored is unclear.

A systematic literature review conducted by the Australian Safety and Efficacy Register of New Interventional Procedures-Surgical[22] identified only six publications related to how acute healthcare organisations introduce new invasive procedures. These included retrospective case-reports of new procedures being introduced in individual hospitals in the UK[23] and Australia,[24] and case studies of qualitative interviews with surgeons and clinicians at hospitals in Canada regarding how decisions to introduce new technologies were made.[25, 26] Findings indicated that the introduction of new procedures and technologies were based predominantly on surgeons' perceptions that such innovations would improve patient outcomes, safety and care, with no structured decision-making process in place at an institutional/governance level. To date, there has been no comprehensive review of current local NHS policies, many of which are inaccessible by traditional literature systematic searches used in the above review.

Aim: To undertake an in-depth analysis of local NHS policies for the introduction of new invasive procedures and devices to establish: I) how policies outline scope for their use, including how they define which invasive procedures and devices are eligible under their remit (e.g. new or modified) and guidance given about when research approvals should be sought, II) recommendations for patient information provision, III) processes for monitoring and reporting outcomes of innovative procedures and devices, including how decision-making regarding adoption or stopping of the procedure or device are made.

Methods and analysis

For peer review only - http://bmjopen.bmj.com/site/about/guidelines.xhtml

For peer review only

This work will comprise two parts occurring concurrently and iteratively - 1) Systematic analysis of written local NHS policies for the introduction of new invasive procedures and devices; 2) Interviews with key stakeholders (e.g. medical directors, audit and governance leads, and surgeons) regarding surgical innovation in practice, including knowledge of and implementation of current policies.

1. Systematic analysis of NHS policies for the introduction of new invasive procedures and devices

Sampling and data collection

All acute NHS trusts in England (n=150) and NHS Health Boards in Wales (n=7)[27] will be systematically approached. Initially, online searches will be performed to determine if policies for the introduction of new invasive procedures and/or devices into clinical practice are available online. Where policies are not available, local NHS organisations will be approached as follows. Medical Directors will be emailed by JMB or RH to request copies of written policies. Non-responders to the initial email will have one email reminder two weeks later and will then be approached by email and phone by a senior research associate and a research fellow. Trusts/health boards not responding to this will have one final email from the original project lead. Policies not wholly related to the introduction of new invasive procedures and/or devices will be excluded (e.g. device management policies, National or Local Safety Standards for Invasive Procedures (NatSSIPs, LocSSIPs)). Invasive procedures will be defined as procedures where access is gained via an incision, natural orifice or percutaneous puncture or involving devices used inside the body.

Trust/health board demographics, including geographical area and acute trust type (England only, e.g. small, specialist, teaching, foundation status) will be collected.[27]

Data extraction from trust policies

For peer review only - http://bmjopen.bmj.com/site/about/guidelines.xhtml

A data extraction form will be developed using a priori themes drawn from the literature and knowledge of the area amongst team members, and themes that inductively emerge from the initial coding of a subset of documents independently by two researchers.

The form will be piloted on a sample of policies gathered during scoping work until the team is satisfied that it fully captures all initial key themes. The finalised form will be converted into an electronic database[28] where data will be inputted directly from written policies. All policy documents, including addendums, will be systematically examined. Ten percent of policies will be randomly selected for double independent data extraction to maximise reliability of data extraction. Where there are discrepancies these will be resolved through consensus and where this is not achieved a third independent reviewer will be consulted.

Data will be extracted about, I) policy scope, II) patient information provision and consent, and III) outcome monitoring and decision making (detailed below). For each policy we will also extract details of policy title and date, date of last policy review and date of next policy review. In addition, if a formal committee is named in the policy as being involved in reviewing the introduction of a new procedure, we will collect data on the title of the committee, committee chair, core members, and frequency of meetings.

I) Policy scope

How policies define which invasive procedures and devices are eligible under their remit (e.g. policy definitions of new procedures) and guidance regarding how to decide whether the new invasive procedure/device requires 'research approvals', in addition to or instead of local policy approvals alone, will be captured. Policy scope data will not be used to inform inclusion or exclusion of policies from analysis.

II) Patient information provision and consent

For peer review only - http://bmjopen.bmj.com/site/about/guidelines.xhtml

Guidance about specific consent procedures and how patients should be informed when a procedure is new, modified or being conducted by a clinician/in a trust for the first time will be captured. Specific requirements relating to Patient Information Leaflets (PILs), the submission of these PILs to the trust for evaluation and any processes in place to monitor adherence to guidance regarding patient information will also be extracted.

III) Outcome monitoring and decision making

In addition to outcomes typically associated with clinical effectiveness studies, outcomes of specific relevance to evaluating innovation of invasive procedures and devices will be extracted; these may include operator experiences, intended function (e.g. benefit) of the new/modified procedure/device, unanticipated harms, and failure and/or abandonment of the procedure/device. Mechanisms proposed for outcome monitoring (e.g. registered audit, feedback to the committee) will be captured, in addition to guidance for decision-making regarding when procedures should be introduced into routine clinical practice or abandoned, and recommendations for wider reporting.

Data analyses (systematic analysis of NHS trust policies)

In order to gain an overview of the scope of the policies and develop an in-depth understanding of specific themes of relevance to the study aims, mixed methods analyses will be undertaken.

Trust/health board demographics, including geographical area and acute trust type will be tabulated. Quantitative data including response rates, and the presence or absence of a new invasive procedures/devices policy within each local NHS organisation will be presented.

Data related to each of the three key data extraction areas outlined above will be tabulated and descriptive statistics provided. For example, the number of policies that provide guidance for when procedures should only be conducted within a research study will be counted.

For peer review only - http://bmjopen.bmj.com/site/about/guidelines.xhtml

Free text data related to each of the three data extraction areas will be extracted from policies and analysed as following. Verbatim sections of policies will be transferred to qualitative data analysis software (NVivo, version 11) and analysed thematically in several key stages: I) two researchers will independently code a subset of policy documents to develop a preliminary coding frame, II) any discrepancies will be resolved through discussion before the coding frame is applied to the full dataset, III) codes will be grouped into themes and subthemes by examining commonalities, differences and relationships in the data, IV) themes will be regularly reviewed to ensure they accurately encapsulate the data. Findings from this qualitative analysis will be written up descriptively.

2. Interviews with key stakeholders

In-depth semi-structured interviews will be conducted with representatives from key stakeholder groups as detailed below.

Recruitment and sampling

Professionals involved in local governance processes related to the introduction of new procedures and devices (e.g. new procedures committee members, medical directors) and healthcare professionals with experience of introducing new/modified procedures or devices into clinical practice (e.g. surgeons, nurses) will be identified from policy documents, trust websites and clinical contacts of the research team. A snowball sampling approach,[29] whereby interviewees are asked to recommend the names of other potential interviewees, will be used to facilitate recruitment. To ensure maximum variation within the sample,[29] we will interview participants from varying geographical locations, trust types, different surgical specialities and from trusts with and without policies. Participant characteristics will be reviewed as recruitment and analyses are ongoing throughout the study, and underrepresented groups or individuals with particular knowledge and/or experiences of particular interest will be purposively sampled.[29] It is anticipated that up to 60

For peer review only - http://bmjopen.bmj.com/site/about/guidelines.xhtml

stakeholders will be interviewed, although data analysis will be driven by the objective of achieving data saturation (whereby no new themes emerge from the data).

Data collection

Written informed consent will be obtained from all participants before interviews commence. Interviews will be conducted by one of two experienced qualitative researchers, either via telephone or in person, at times convenient for participants. Interviews will be audio-recorded using encrypted audio-recording devices. Discussions will follow a topic guide that will vary by stakeholder group so that key issues are covered, while ensuring participants are able to talk about new issues they feel are important. Topic guides will be adapted iteratively as analyses of interviews and written policies progresses so that any emerging issues can be discussed with subsequent participants.

Interviews with professionals involved in governance processes will specifically explore how new procedures are introduced. This will include how policies define the types of technologies and procedures that will be reviewed for introduction to the trust (with examples if possible); the approval processes in place; methods for follow up, monitoring of approved technologies/procedures and abandonment of procedures. Interviews with healthcare professionals will explore their views on surgical innovation, monitoring of new procedures/devices and, when applicable, their experience of introducing a new procedure or device into clinical practice.

Analysis (interviews with key stakeholders)

Interviews will be transcribed in full and verbatim, checked against the original recording for accuracy, and imported into NVivo (version 11). Data will then be systematically assigned codes and analysed thematically using constant comparative techniques.[30] A subset of the transcripts will be doubled coded by a second qualitative researcher, with any discrepancies in coding discussed and resolved. Data collection and analysis will proceed in parallel, with emerging findings informing further sampling (theoretical sampling) and data collection. Dissonant views that challenge the

For peer review only - http://bmjopen.bmj.com/site/about/guidelines.xhtml

emerging dominant perspectives will be actively pursed (negative case analysis) to ensure the inclusion of diverse viewpoints.

Data protection and confidentiality

All data relating to participants' personal identities will be anonymised using unique study identifiers. This data will be stored in a separate encrypted file, in a separate location from the study data on the University of Bristol server. It will only be accessible to the research team and used only in the event of re-contacting study participants to verify information, e.g. quotes. Verbatim quotations that may be used for publications or presentations will also be anonymised.

Ethics and dissemination

The in-depth analysis of written policies does not require ethics approval, in accordance with the HRA definition of research.[31] The qualitative component of the work has been approved by the University of Bristol Ethics Committee (reference 56522).

Expected outcomes of the study

This work will provide an in-depth exploration and summary of current governance procedures for clinicians wishing to introduce innovative invasive procedures and devices into NHS practice in England and Wales. Understanding how innovative invasive procedures are introduced will inform the development of standardised guidance and raise hypotheses for future research. It is expected that this work will inform national guidelines regarding the introduction of innovative invasive procedures.

Dissemination

Findings from this work will be presented at appropriate conferences and published across several papers highlighting main findings. These will include in-depth analyses of the scope of written policies, including when innovative procedures should be delivered within a research governance framework, and how policies define 'new' and 'modified' procedures/devices. Additional

For peer review only - http://bmjopen.bmj.com/site/about/guidelines.xhtml

For peer review only

publications will focus on guidance related to patient information, consent and outcome monitoring after the introduction of new procedures and devices. Additionally, qualitative data from interviews with key stakeholders regarding surgical innovation in practice, including knowledge and implementation of current policies will be published separately.

Discussion

Currently little is known about the ways in which new invasive procedures and devices are introduced into NHS clinical practice outside the context of research. This is a topical issue as several concerning and problematic high-profile cases have emerged recently.[5-7, 32] This study will systematically study current NHS practice to determine the presence, content and implementation of policies relating to the introduction of new invasive procedures and devices in NHS England and Wales. Identified policies will be scrutinised to determine what guidance is given regarding policy scope, patient information provision and the monitoring, reporting and review of outcomes. Additionally, interviews with stakeholders (such as surgeons and members of new procedures committees) will further inform this work.

The ways in which new procedures, including surgery, are introduced into practice is a topic of international public interest. This study is the first of its kind and it is anticipated it will inform future NHS governance and practice in this field.

Patient and public involvement

The current study comprises a core component of the work undertaken within the National Institute for Health Research (NIHR) Bristol Biomedical Research Centre (BRC) Surgical Innovation theme, which aims to improve the safe and transparent translation of innovative procedures/devices to clinical practice. A patient and public involvement (PPI) group has been established as part of the NIHR Bristol BRC, where patients who have undergone surgery are asked about their views regarding how new surgical procedures are undertaken in NHS clinical practice. This involves discussion around

For peer review only - http://bmjopen.bmj.com/site/about/guidelines.xhtml

what information patients would like to be provided with before and after receiving a new invasive procedure/device, and what health/lifestyle outcomes after surgery would be considered important. To date the consensus between PPI group members is that the work being undertaken to improve the way in which new procedures are introduced into clinical practice is important and could have positive implications for future health care in the NHS. The current study comprises a first step in the process of improving how new procedures are introduced into practice and the PPI group will continue to provide input throughout the study and future work to develop related guidance and disseminate findings.

Authors' contributions

All authors contributed to the development of the idea and drafting and revision of the manuscript. JMB is the lead of the NIHR Bristol Biomedical Research Centre Surgical Innovation theme and formed the methodological ideas to understand surgical innovation with contributions from SC, HR, JZ, DE, KA, SP, NW, BGM, NSB, RH. All authors gave approval for the manuscript to be submitted.

Funding statement

This study was supported by the NIHR Biomedical Research Centre at University Hospitals Bristol NHS Foundation Trust and the University of Bristol, the MRC ConDuCT-II (Collaboration and innovation for Difficult and Complex randomised controlled Trials In Invasive procedures) Hub for Trials Methodology Research (MR/K025643/1) (www.bristol.ac.uk/population-health-sciences/centres/conduct2) and a NIHR senior investigator award (NF-SI-0514-10114). The views expressed in this publication are those of the author(s) and not necessarily those of the NHS, the National Institute for Health Research, the Department of Health and Social Care or the MRC.

Competing interests statement

None declared

References

For peer review only

1. Weiser TG, Regenbogen SE, Thompson KD, et al. An estimation of the global volume of surgery: a modelling strategy based on available data. *Lancet* 2008;372(9633):139-44. doi: 10.1016/S0140-6736(08)60878-8 [published Online First: 2008/06/28]
2. Abbott TEF, Fowler AJ, Dobbs TD, et al. Frequency of surgical treatment and related hospital procedures in the UK: a national ecological study using hospital episode statistics. *Br J Anaesth* 2017;119(2):249-57. doi: 10.1093/bja/aex137 [published Online First: 2017/09/01]
3. Poelman M, Apers J, van den Brand H, et al. The INCH-Trial: a multicentre randomized controlled trial comparing the efficacy of conventional open surgery and laparoscopic surgery for incisional hernia repair. *BMC surgery* 2013;13:18-18. doi: 10.1186/1471-2482-13-18
4. Edwards TL, Xue K, Meenink HCM, et al. First-in-human study of the safety and viability of intraocular robotic surgery. *Nat Biomed Eng* 2018;2:649-56. doi: 10.1038/s41551-018-0248-4 [published Online First: 2018/09/29]
5. Cohen D. How a fake hip showed up failings in European device regulation. *BMJ* 2012;345:e7090. doi: 10.1136/bmj.e7090 %J
6. Dyer C. Robot assisted surgery is blamed for heart patient's death. *BMJ* 2018;363:k4791. doi: 10.1136/bmj.k4791 %J
7. Heneghan C, Aronson JK, Goldacre B, et al. Transvaginal mesh failure: lessons for regulation of implantable devices. *BMJ* 2017;359:j5515. doi: 10.1136/bmj.j5515 [published Online First: 2017/12/09]
8. Royal College of Surgeons. RCS statement on need for national guidelines on the introduction of new procedures and technologies 2018 [Available from: https://www.rcseng.ac.uk/news-and-events/media-centre/press-releases/robotic-surgery-guidelines/ accessed November 2018]
9. Blencowe NS, Chana P, Whistance RN, et al. Outcome reporting in neoadjuvant surgical trials: a systematic review of the literature and proposals for new standards. *J Natl Cancer Inst* 2014;106(9) doi: 10.1093/jnci/dju217 [published Online First: 2014/08/12]
10. Blencowe NS, McNair AG, Davis CR, et al. Standards of outcome reporting in surgical oncology: a case study in esophageal cancer. *Ann Surg Oncol* 2012;19(13):4012-8. doi: 10.1245/s10434-012-2497-x [published Online First: 2012/07/24]
11. Currie A, Brigic A, Blencowe NS, et al. Systematic review of surgical innovation reporting in laparoendoscopic colonic polyp resection. *Br J Surg* 2015;102(2):e108-16. doi: 10.1002/bjs.9675 [published Online First: 2015/01/30]
12. Whistance RN, Forsythe RO, McNair AG, et al. A systematic review of outcome reporting in colorectal cancer surgery. *Colorectal Dis* 2013;15(10):e548-60. doi: 10.1111/codi.12378 [published Online First: 2013/08/10]
13. Directive 2000/70/EC of the European Parliament and of the Council of 16 November 2000 amending Council Directive 93/42/EEC as regards medical devices incorporating stable derivates of human blood or human plasma [Availiable from: https://eur-lex.europa.eu/legal-content/en/TXT/?uri=CELEX:32000L0070 accessed December 2018]
14. Council Directive 90/385/EEC of 20 June 1990 on the approximation of the laws of the Member States relating to active implantable medical devices 1990;189(20/07):0017-36.
15. Kramer DB, Xu S, Kesselheim AS. Regulation of medical devices in the United States and European Union: Mass Medical Soc, 2012.
16. Legislation.gov.uk. The Human Medicines Regulations 2012 2012 [Available from: http://www.legislation.gov.uk/uksi/2012/1916/regulation/8/made accessed February 2018]
17. National Institute for Health and Care Excellence. Interventional procedures guidance 2003 [Available from: https://www.nice.org.uk/about/what-we-do/our-programmes/nice-guidance/nice-interventional-procedures-guidance accessed February 2018]
18. Department of Health. Health Service Circular (HSC2003/011) – The Interventional Procedures Programme 2003 [Available from: https://webarchive.nationalarchives.gov.uk/20120503190439/http://www.dh.gov.uk/prod_

For peer review only - http://bmjopen.bmj.com/site/about/guidelines.xhtml

consum_dh/groups/dh_digitalassets/@dh/@en/documents/digitalasset/dh_4064925.pdf accessed February 2018]
19. Biffl WL, Spain DA, Reitsma AM, et al. Responsible development and application of surgical innovations: a position statement of the Society of University Surgeons. *JACS* 2008;206(6):1204-09.
20. Stefanidis D, Fanelli RD, Price R, et al. SAGES guidelines for the introduction of new technology and techniques. *Surg Endosc* 2014;28(8):2257-71. doi: 10.1007/s00464-014-3587-6 [published Online First: 2014/06/19]
21. Royal College of Surgeons. Good Surgical Practice - Introduction of new techniques 2014 [Available from: https://www.rcseng.ac.uk/standards-and-research/gsp/domain-1/1-2-4-introduction-of-new-techniques/ accessed February 2018]
22. Thavaneswaran P, Maddern G, Spigelman A, et al. A review of policies and processes for the introduction of new interventional procedures. ASERNIP-S Report No. 58. Adelaide, South Australia: ASERNIP, 2007.23.
23. Freedman DB, Swanson E. Introducing new interventional procedures within a District General Hospital, linking with the process for implementing NICE guidance. *Clinical Governance: An International Journal* 2006;11(3):180-86. doi: doi:10.1108/14777270610683100
24. Spigelman AD. Governance and innovation: experience with a policy on the introduction of new interventional procedures. *ANZ J Surg* 2006;76(1-2):9-13. doi: 10.1111/j.1445-2197.2006.03648.x [published Online First: 2006/02/18]
25. Danjoux NM, Martin DK, Lehoux PN, et al. Adoption of an innovation to repair aortic aneurysms at a Canadian hospital: a qualitative case study and evaluation. *BMC Health Serv Res* 2007;7:182. doi: 10.1186/1472-6963-7-182 [published Online First: 2007/11/17]
26. Sharma B, Danjoux NM, Harnish JL, et al. How are decisions to introduce new surgical technologies made? Advanced laparoscopic surgery at a Canadian community hospital: A qualitative case study and evaluation. *Surg Innov* 2006;13(4):250-6. doi: 10.1177/1553350606296341 [published Online First: 2007/01/18]
27. NHS Digital. Estates Return Information Collection, England, 2016-2017 2017 [Available from: https://digital.nhs.uk/data-and-information/publications/statistical/estates-returns-information-collection/estates-return-information-collection-2016-17 accessed February 2018]
28. JISC. Online Surveys 2017 [Available from: https://www.onlinesurveys.ac.uk accessed April 2018]
29. Bryman A. Social research methods: Oxford university press 2016.
30. Braun V, Clarke, V., & Terry, G. . Thematic Analysis. In: Rohleder PL, A., ed. Qualitative research in clinical and health psychology. Basingstoke: Palgrave Macmillan 2014.
31. Health Research Authority. HRA Defining Research 2017 [Available from: http://www.hra-decisiontools.org.uk/research/docs/DefiningResearchTable_Oct2017-1.pdf accessed February 2018]
32. Gornall J. The trial that launched millions of mesh implant procedures: did money compromise the outcome? *BMJ* 2018;363:k4155. doi: 10.1136/bmj.k4155 %J

For peer review only - http://bmjopen.bmj.com/site/about/guidelines.xhtml

BMJ Open

The introduction and adoption of innovative invasive procedures and devices in the NHS: an in-depth analysis of written policies and qualitative interviews (the INTRODUCE study protocol)

Journal:	*BMJ Open*
Manuscript ID	bmjopen-2019-029963.R1
Article Type:	Protocol
Date Submitted by the Author:	15-Jul-2019

Complete List of Authors:	Cousins, Sian; University of Bristol, Population Health Sciences<br>Richards, Hollie; University of Bristol, Population Health Sciences<br>Zahra, Jesmond; University of Bristol, Population Health Sciences<br>Elliott, Daisy; University of Bristol, Population Health Sciences<br>Avery, Kerry; University of Bristol, Population Health Sciences<br>Robertson, Harry; University of Bristol, Population Health Sciences<br>Paramasivan, Sangeetha ; University of Bristol, Population Health Sciences<br>Wilson, Nicholas; University of Bristol, Population Health Sciences<br>Mathews, Johnny; University of Bristol, Population Health Sciences<br>Tolkien, Zoe; University of Bristol, Population Health Sciences<br>Main, Barry; University of Bristol, Population Health Sciences; University Hospitals Bristol NHS Foundation Trust<br>Blencowe, Natalie; University of Bristol, Population Health Sciences; University Hospitals Bristol NHS Foundation Trust<br>Hinchliffe, Robert; University of Bristol, Population Health Sciences; North Bristol NHS Trust<br>Blazeby, Jane; University of Bristol, Population Health Sciences; University Hospitals Bristol NHS Foundation Trust

**Primary Subject Heading**:	Surgery
Secondary Subject Heading:	Health services research
Keywords:	SURGERY, innovation, NHS, Clinical governance < HEALTH SERVICES ADMINISTRATION & MANAGEMENT

SCHOLARONE™
Manuscripts

The introduction and adoption of innovative invasive procedures and devices in the NHS: an in-depth analysis of written policies and qualitative interviews (the INTRODUCE study protocol)

Cousins S,[1] Richards H,[1] Zahra J,[1] Elliott D,[1] Avery K,[1] Robertson HF,[1] Paramasivan S,[1] Wilson N,[1] Mathews J,[1] Tolkien Z,[1] Main BG,[1,2] Blencowe NS,[1,2] Hinchliffe R,[1,3] Blazeby JM[1,2]

[1]National Institute for Health Research Bristol Biomedical Research Centre Surgical Innovation Theme and the Medical Research Council ConDuCT-II (Collaboration and innovation in Difficult and Complex randomised controlled Trials In Invasive procedures) Hub for Trials Methodology Research, Centre for Surgical Research, Population Health Sciences, Bristol Medical School, University of Bristol, 39 Whatley Road, Clifton, Bristol, UK. BS8 2PS;

[2]University Hospitals Bristol NHS Foundation Trust, Bristol, UK. BS2 8HW

[3]North Bristol NHS Trust, Bristol, UK. BS10 5NB

Corresponding author: Sian Cousins, Sian.cousins@bristol.ac.uk

Word count: 3089 (4000 max)

For peer review only - http://bmjopen.bmj.com/site/about/guidelines.xhtml

Abstract

Introduction

Innovation is key to improving outcomes in healthcare. Innovative pharmaceutical products undergo rigorous phased research evaluation before they are introduced into practice. The introduction of innovative invasive procedures and devices is much less rigorous and phased research, including randomised controlled trials, is not always undertaken. Whilst the innovator (usually a surgeon) may introduce a new or modified procedure/device within the context of formal research, they may also be introduced by applying for local NHS organisation approval alone. Written policies for the introduction of new procedures and/or devices often form part of this local clinical governance infrastructure; however, little is known about their content or use in practice.

This study aims to systematically investigate how new invasive procedures and devices are introduced in NHS England and Wales.

Methods and analysis

An in-depth analysis of written policies will be undertaken. This will be supplemented with interviews with key stakeholders.

All acute NHS trusts in England and Health Boards in Wales will be systematically approached and asked to provide written policies for the introduction of new invasive procedures and devices. Information on the following will be captured: I) policy scope, including when new procedures should be introduced within a formal research framework; II) requirements for patient information provision; III) outcome reporting and/or monitoring. Data will be extracted using a standardised form developed iteratively within the study team.

Semi-structured interviews with medical directors, audit and governance leads, and surgeons will explore views regarding the introduction of new invasive procedures into practice, including knowledge of and implementation of current policies.

For peer review only

For peer review only - http://bmjopen.bmj.com/site/about/guidelines.xhtml

Ethics and dissemination

In-depth analysis of written policies does not require ethics approval. The University of Bristol Ethics Committee (56522) approved the interview component of the study. Findings from this work will be presented at appropriate conferences and will be published in peer-reviewed journals.

Article summary

Strengths and limitations of this study

- This is the first systematic study addressing how the NHS introduces innovative invasive procedures into clinical practice; a topic of international public, professional and political interest given the wealth of historic and recent examples of patient harm caused by lack of regulation.
- The study combines analysis of written policies and interviews with key stakeholders to ensure an in-depth exploration of the topic to inform national guidance to improve and standardise the introduction of innovative invasive procedures and devices.
- The degree to which policies are actually adhered to across local NHS organisations is not systematically explored in the current study and is recommended for future research.

Introduction

Invasive procedures are a fundamental part of healthcare and can include surgical operations with and without devices, as well as endoscopic and radiologically guided interventions. At least 230 million invasive procedures are delivered worldwide,[1] with 12.5 million undertaken in the United Kingdom (UK) annually.[2] This number is likely to increase with continued innovation, including the advent of new technologies and minimal access procedures.

Innovation in invasive procedures is key. It may include modifying existing techniques,[3] to performing completely new first-in-human invasive procedures.[4] Whilst innovation is common, the governance surrounding it is not standardised and is currently under much scrutiny.[5-7] A recent

For peer review only - http://bmjopen.bmj.com/site/about/guidelines.xhtml

inquest into the death of a patient following robot-assisted cardiac surgery, a procedure that had not been previously performed in the UK, found insufficient governance surrounding the introduction of this innovative procedure.[6] Recommendations from the Coroner, echoed by a statement from RCS,[8] included introducing stricter governance relating to the use of new technologies and procedures, specific measures to assess the competence and training received by clinicians wishing to undertake them, and detailed patient information provision regarding the risks associated with new procedures.

Innovation in invasive procedures may be introduced under the auspices of formal research studies, with a protocol and application for ethical approval. However, multiple reviews show that ethical approval is rarely gained when delivering innovative invasive procedures.[9-12] While medical devices to be used inside the body require a European Conformity (CE) mark[13-15] prior to use in the UK, the evidence required to gain this certification does not often come from high-quality randomised controlled trials and post-marketing surveillance is minimal.[7] Although medical device regulations are improving,[16] this is very different to the tightly governed and transparent developmental pathways required for the introduction of new pharmaceuticals, including requirements for formal assessments of risk-benefit balance and post-market safety monitoring.[17]

Outside of research, innovative invasive procedures and devices may be introduced via local hospital policies. The UK National Institute for Clinical Excellence (NICE) is an independent body responsible for providing evidence-based guidance to the UK National Health Service (NHS) on health and social care. The NHS refers to the four publicly funded healthcare services in the UK (NHS in England, NHS Wales, NHS Scotland and Health and Social care in Northern Ireland) and is made up of local NHS organisations (e.g. NHS trusts in England and health boards in Wales) (supplementary file 1). It is recommended by NICE that these local NHS organisations have appropriate governance structures in place to review, authorise and monitor the introduction of new invasive procedures.[18] In addition, NICE recommends the approval of new invasive procedures that do not have existing NICE guidance

For peer review only - http://bmjopen.bmj.com/site/about/guidelines.xhtml

should only be given if appropriate training of those delivering the procedure is demonstrated, patients are made aware of the new status of the procedures, and there are proposed arrangements for clinical audit.[19] These recommendations are echoed by organisations worldwide.[20-22] In the UK, it is the responsibility of local hospital organisations to implement this guidance, although the extent to which this occurs is unknown, and there are instances where innovative procedures are introduced into practice without any formal governance.[6] Furthermore, where local policies do exist, little is known about their content or how they are used in practice; how trusts define a new procedure (i.e. when the guidance should be applied), what information should be given to patients, and how outcomes of new procedures are recorded and monitored is unclear. Examination of when policies are being applied provides valuable information about the presence or absence of local governance frameworks for the introduction of innovative invasive procedures. Furthermore, guidance related to patient information provision will provide insight into whether patients are informed about the innovative status of procedures to be delivered. It is also important that outcomes are routinely and effectively monitored to support their continued use or to ensure those that are ineffective and/or unsafe are abandoned.

A systematic literature review conducted by the Australian Safety and Efficacy Register of New Interventional Procedures-Surgical[23] identified only six publications related to how acute healthcare organisations introduce new invasive procedures. These included retrospective case-reports of new procedures being introduced in individual hospitals in the UK[24] and Australia,[25] and case studies of qualitative interviews with surgeons and clinicians at hospitals in Canada regarding how decisions to introduce new technologies were made.[26 27] Findings indicated that the introduction of new procedures and technologies were based predominantly on surgeons' perceptions that such innovations would improve patient outcomes, safety and care, with no structured decision-making process in place at an institutional/governance level. To date, there has been no comprehensive review of current local NHS policies, many of which are inaccessible by traditional literature systematic searches used in the above review.

For peer review only - http://bmjopen.bmj.com/site/about/guidelines.xhtml

Aim: To undertake an in-depth analysis of local NHS policies to establish the governance in place for the introduction of new invasive procedures and devices. This will include examination of: I) how policies outline scope for their use, including how they define which invasive procedures and devices are eligible under their remit (e.g. new or modified) and guidance given about when research approvals should be sought, II) recommendations for patient information provision, III) processes for monitoring and reporting outcomes of innovative procedures and devices, including how decision-making regarding adoption or stopping of the procedure or device are made.

Methods and analysis

This work will comprise two parts occurring concurrently and iteratively - 1) Systematic analysis of written local NHS policies for the introduction of new invasive procedures and devices; 2) Interviews with key stakeholders (e.g. medical directors, audit and governance leads, and surgeons) regarding surgical innovation in practice, including knowledge of and implementation of current policies.

1. Systematic analysis of NHS policies for the introduction of new invasive procedures and devices

Sampling and data collection

All acute NHS trusts in England (n=150) and NHS Health Boards in Wales (n=7)[27] will be systematically approached (Figure 1). Initially, online searches will be performed to determine if policies for the introduction of new invasive procedures and/or devices into clinical practice are available online. An online search engine (Google) will be used to locate the website for each NHS trust/health board. The individual trust/HB website search function will be used to determine if the organisation has a copy of the relevant policy available online using search terms such as ‘new procedure’, ‘policy/policies’. Exploratory searching of the trust/HB website will also be conducted to ensure relevant information is not missed. Where policies are not available, local NHS organisations will be approached as follows. Medical Directors will be emailed by senior authors (JMB or RH) to

For peer review only - http://bmjopen.bmj.com/site/about/guidelines.xhtml

request copies of written policies. Non-responders to the initial email will have one email reminder two weeks later and will then be approached by email and phone by a senior research associate and a research fellow. Trusts/health boards not responding to this will have one final email from the original project lead. Policies not wholly related to the introduction of new invasive procedures and/or devices will be excluded (e.g. device management policies, National or Local Safety Standards for Invasive Procedures (NatSSIPs, LocSSIPs)). Invasive procedures will be defined as procedures where access is gained via an incision, natural orifice or percutaneous puncture or involving devices used inside the body.

Trust/health board demographics, including geographical area and acute trust type (England only, e.g. small, specialist, teaching, foundation status) will be collected.[28]

Data extraction from trust policies

A data extraction form will be developed using a priori themes drawn from the literature and knowledge of the area amongst team members (see supplementary file 2 for a preliminary data extraction form), and themes that inductively emerge from the initial coding of a subset of documents independently by two researchers.

The form will be piloted on a sample of policies gathered during scoping work until the team is satisfied that it fully captures all initial key themes. The finalised form will be converted into an electronic database[29] where data will be inputted directly from written policies. All policy documents, including addendums, will be systematically examined. Ten percent of policies will be randomly selected for double independent data extraction to maximise reliability of data extraction. Where there are discrepancies these will be resolved through consensus and where this is not achieved a third independent reviewer will be consulted.

Data will be extracted about, I) policy scope, II) patient information provision and consent, and III) outcome monitoring and decision making (detailed below). For each policy we will also extract

For peer review only - http://bmjopen.bmj.com/site/about/guidelines.xhtml

details of policy title and date, date of last policy review and date of next policy review. In addition, if a formal committee is named in the policy as being involved in reviewing the introduction of a new procedure, we will collect data on the title of the committee, committee chair, core members, and frequency of meetings.

I) Policy scope

How policies define which invasive procedures and devices are eligible under their remit (e.g. policy definitions of new procedures) and guidance regarding how to decide whether the new invasive procedure/device requires 'research approvals', in addition to or instead of local policy approvals alone, will be captured. Policy scope data will not be used to inform inclusion or exclusion of policies from analysis.

II) Patient information provision and consent

Guidance about specific consent procedures and how patients should be informed when a procedure is new, modified or being conducted by a clinician/in a trust for the first time will be captured. Specific requirements relating to Patient Information Leaflets (PILs), the submission of these PILs to the trust for evaluation and any processes in place to monitor adherence to guidance regarding patient information will also be extracted.

III) Outcome monitoring and decision making

In addition to outcomes typically associated with clinical effectiveness studies, outcomes of specific relevance to evaluating innovation of invasive procedures and devices will be extracted; these may include operator experiences, intended function (e.g. benefit) of the new/modified procedure/device, unanticipated harms, and failure and/or abandonment of the procedure/device. Mechanisms proposed for outcome monitoring (e.g. registered audit, feedback to the committee) will be captured, in addition to guidance for decision-making

For peer review only - http://bmjopen.bmj.com/site/about/guidelines.xhtml

regarding when procedures should be introduced into routine clinical practice or abandoned, and recommendations for wider reporting.

Data analyses (systematic analysis of NHS trust policies)

In order to gain an overview of the scope of the policies and develop an in-depth understanding of specific themes of relevance to the study aims, mixed methods analyses will be undertaken.

Trust/health board demographics, including geographical area and acute trust type will be tabulated. Quantitative data including response rates, and the presence or absence of a new invasive procedures/devices policy within each local NHS organisation will be presented.

Data related to each of the three key data extraction areas outlined above will be tabulated and descriptive statistics provided. For example, the number of policies that provide guidance for when procedures should only be conducted within a research study will be counted.

Free text data related to each of the three data extraction areas will be extracted from policies and analysed as following. Verbatim sections of policies will be transferred to qualitative data analysis software (NVivo, version 11) and analysed thematically in several key stages: I) two researchers will independently code a subset of policy documents to develop a preliminary coding frame, II) any discrepancies will be resolved through discussion before the coding frame is applied to the full dataset, III) codes will be grouped into themes and subthemes by examining commonalities, differences and relationships in the data, IV) themes will be regularly reviewed to ensure they accurately encapsulate the data. Findings from this qualitative analysis will be written up descriptively.

2. Interviews with key stakeholders

In-depth semi-structured interviews will be conducted with representatives from key stakeholder groups as detailed below.

For peer review only - http://bmjopen.bmj.com/site/about/guidelines.xhtml

Recruitment and sampling

Professionals involved in local governance processes related to the introduction of new procedures and devices (e.g. new procedures committee members, medical directors) and healthcare professionals with experience of introducing new/modified procedures or devices into clinical practice (e.g. surgeons, nurses) will be identified from policy documents, trust websites and clinical contacts of the research team. A snowball sampling approach,[30] whereby interviewees are asked to recommend the names of other potential interviewees, will be used to facilitate recruitment. To ensure maximum variation within the sample,[30] we will interview participants from varying geographical locations, trust types, different surgical specialities and from trusts with and without policies. Participant characteristics will be reviewed as recruitment and analyses are ongoing throughout the study, and underrepresented groups or individuals with particular knowledge and/or experiences of particular interest will be purposively sampled.[30] It is anticipated that up to 60 stakeholders will be interviewed, although data analysis will be driven by the objective of achieving data saturation (whereby no new themes emerge from the data).

Data collection

Written informed consent will be obtained from all participants before interviews commence. Interviews will be conducted by one of two experienced qualitative researchers, either via telephone or in person, at times convenient for participants. Interviews will be audio-recorded using encrypted audio-recording devices (Olympus DS3500). Discussions will follow a topic guide that will vary by stakeholder group so that key issues are covered, while ensuring participants are able to talk about new issues they feel are important. Topic guides (see supplementary file 3 for example preliminary topic guide for clinicians) will be adapted iteratively as analyses of interviews and written policies progresses so that any emerging issues can be discussed with subsequent participants.

Interviews with professionals involved in governance processes will specifically explore how new procedures are introduced. This will include how policies define the types of technologies and

For peer review only - http://bmjopen.bmj.com/site/about/guidelines.xhtml

procedures that will be reviewed for introduction to the trust (with examples if possible); the approval processes in place; methods for follow up, monitoring of approved technologies/procedures and abandonment of procedures. Interviews with healthcare professionals will explore their views on surgical innovation, monitoring of new procedures/devices and, when applicable, their experience of introducing a new procedure or device into clinical practice.

Analysis (interviews with key stakeholders)

Interviews will be transcribed in full and verbatim, checked against the original recording for accuracy, and imported into NVivo (version 11). Data will then be systematically assigned codes and analysed thematically using constant comparative techniques.[31] A subset of the transcripts will be doubled coded by a second qualitative researcher, with any discrepancies in coding discussed and resolved. Data collection and analysis will proceed in parallel, with emerging findings informing further sampling (theoretical sampling) and data collection. Dissonant views that challenge the emerging dominant perspectives will be actively pursed (negative case analysis) to ensure the inclusion of diverse viewpoints. Descriptive accounts of the data, which take into consideration of the views and background of the analysts, will then be written.

Data protection and confidentiality

All data relating to participants' personal identities will be anonymised using unique study identifiers. This data will be stored in a separate encrypted file, in a separate location from the study data on the University of Bristol server. It will only be accessible to the research team and used only in the event of re-contacting study participants to verify information, e.g. quotes. Verbatim quotations that may be used for publications or presentations will also be anonymised.

Ethics and dissemination

For peer review only - http://bmjopen.bmj.com/site/about/guidelines.xhtml

The in-depth analysis of written policies does not require ethics approval, in accordance with the HRA definition of research.[32] The qualitative component of the work has been approved by the University of Bristol Ethics Committee (reference 56522).

Expected outcomes of the study

This work will provide an in-depth exploration and summary of current governance procedures for clinicians wishing to introduce innovative invasive procedures and devices into NHS practice in England and Wales. Understanding how innovative invasive procedures are introduced will identify limitations in current guidance, inform the development of standardised guidance and raise hypotheses for future research. It is expected that this work will inform national guidelines regarding the introduction of innovative invasive procedures.

Dissemination

Findings from this work will be presented at appropriate conferences and published across several papers highlighting main findings. These will include in-depth analyses of the scope of written policies, including when innovative procedures should be delivered within a research governance framework, and how policies define 'new' and 'modified' procedures/devices. Additional publications will focus on guidance related to patient information, consent and outcome monitoring after the introduction of new procedures and devices. Additionally, qualitative data from interviews with key stakeholders regarding surgical innovation in practice, including knowledge and implementation of current policies will be published separately.

Discussion

Currently little is known about the ways in which new invasive procedures and devices are introduced into NHS clinical practice outside the context of research. This is a topical issue as several concerning and problematic high-profile cases have emerged recently.[5-7, 33] This study will systematically study current NHS practice to determine the presence, content and implementation

For peer review only - http://bmjopen.bmj.com/site/about/guidelines.xhtml

of policies relating to the introduction of new invasive procedures and devices in NHS England and Wales. Identified policies will be scrutinised to determine what guidance is given regarding policy scope, patient information provision and the monitoring, reporting and review of outcomes. Additionally, interviews with stakeholders (such as surgeons and members of new procedures committees) will further inform this work.

The ways in which new procedures, including surgery, are introduced into practice is a topic of international public interest. This study is the first of its kind and it is anticipated it will inform future NHS governance and practice in this field.

Patient and public involvement

The current study comprises a core component of the work undertaken within the National Institute for Health Research (NIHR) Bristol Biomedical Research Centre (BRC) Surgical Innovation theme, which aims to improve the safe and transparent translation of innovative procedures/devices to clinical practice. A patient and public involvement (PPI) group has been established as part of the NIHR Bristol BRC, where patients who have undergone surgery are asked about their views regarding how new surgical procedures are undertaken in NHS clinical practice. This involves discussion around what information patients would like to be provided with before and after receiving a new invasive procedure/device, and what health/lifestyle outcomes after surgery would be considered important. To date the consensus between PPI group members is that the work being undertaken to improve the way in which new procedures are introduced into clinical practice is important and could have positive implications for future health care in the NHS. The current study comprises a first step in the process of improving how new procedures are introduced into practice and the PPI group will continue to provide input throughout the study and future work to develop related guidance and disseminate findings.

Authors' contributions

For peer review only - http://bmjopen.bmj.com/site/about/guidelines.xhtml

All authors contributed to the development of the idea and drafting and revision of the manuscript. JMB is the lead of the NIHR Bristol Biomedical Research Centre Surgical Innovation theme and formed the methodological ideas to understand surgical innovation with contributions from SC, HR, JZ, DE, KA, HR, SP, NW, JM, ZT, BGM, NSB, RH. All authors gave approval for the manuscript to be submitted.

Funding statement

This study was supported by the NIHR Biomedical Research Centre at University Hospitals Bristol NHS Foundation Trust and the University of Bristol, the MRC ConDuCT-II (Collaboration and innovation for Difficult and Complex randomised controlled Trials In Invasive procedures) Hub for Trials Methodology Research (MR/K025643/1) (www.bristol.ac.uk/population-health-sciences/centres/conduct2) and a NIHR senior investigator award (NF-SI-0514-10114). The views expressed in this publication are those of the author(s) and not necessarily those of the NHS, the National Institute for Health Research, the Department of Health and Social Care or the MRC.

Competing interests' statement

None declared

References

1. Weiser TG, Regenbogen SE, Thompson KD, et al. An estimation of the global volume of surgery: a modelling strategy based on available data. *Lancet* 2008;372(9633):139-44. doi: 10.1016/S0140-6736(08)60878-8 [published Online First: 2008/06/28]
2. Abbott TEF, Fowler AJ, Dobbs TD, et al. Frequency of surgical treatment and related hospital procedures in the UK: a national ecological study using hospital episode statistics. *Br J Anaesth* 2017;119(2):249-57. doi: 10.1093/bja/aex137 [published Online First: 2017/09/01]
3. Poelman M, Apers J, van den Brand H, et al. The INCH-Trial: a multicentre randomized controlled trial comparing the efficacy of conventional open surgery and laparoscopic surgery for incisional hernia repair. *BMC surgery* 2013;13:18-18. doi: 10.1186/1471-2482-13-18
4. Edwards TL, Xue K, Meenink HCM, et al. First-in-human study of the safety and viability of intraocular robotic surgery. *Nat Biomed Eng* 2018;2:649-56. doi: 10.1038/s41551-018-0248-4 [published Online First: 2018/09/29]
5. Cohen D. How a fake hip showed up failings in European device regulation. 2012;345:e7090. doi: 10.1136/bmj.e7090 %J BMJ : British Medical Journal
6. Dyer C. Robot assisted surgery is blamed for heart patient's death. 2018;363:k4791. doi: 10.1136/bmj.k4791 %J BMJ

For peer review only - http://bmjopen.bmj.com/site/about/guidelines.xhtml

7. Heneghan C, Aronson JK, Goldacre B, et al. Transvaginal mesh failure: lessons for regulation of implantable devices. *BMJ* 2017;359:j5515. doi: 10.1136/bmj.j5515 [published Online First: 2017/12/09]
8. Royal College of Surgeons of England. RCS statement on need for national guidelines on the introduction of new procedures and technologies 2018 [Available from: https://www.rcseng.ac.uk/news-and-events/media-centre/press-releases/robotic-surgery-guidelines/ accessed 09/11/2018.
9. Blencowe NS, Chana P, Whistance RN, et al. Outcome reporting in neoadjuvant surgical trials: a systematic review of the literature and proposals for new standards. *J Natl Cancer Inst* 2014;106(9) doi: 10.1093/jnci/dju217 [published Online First: 2014/08/12]
10. Blencowe NS, McNair AG, Davis CR, et al. Standards of outcome reporting in surgical oncology: a case study in esophageal cancer. *Ann Surg Oncol* 2012;19(13):4012-8. doi: 10.1245/s10434-012-2497-x [published Online First: 2012/07/24]
11. Currie A, Brigic A, Blencowe NS, et al. Systematic review of surgical innovation reporting in laparoendoscopic colonic polyp resection. *Br J Surg* 2015;102(2):e108-16. doi: 10.1002/bjs.9675 [published Online First: 2015/01/30]
12. Whistance RN, Forsythe RO, McNair AG, et al. A systematic review of outcome reporting in colorectal cancer surgery. *Colorectal Dis* 2013;15(10):e548-60. doi: 10.1111/codi.12378 [published Online First: 2013/08/10]
13. Directive 2000/70/EC of the European Parliament and of the Council of 16 November 2000 amending Council Directive 93/42/EEC as regards medical devices incorporating stable derivates of human blood or human plasma [Availiable from: https://eur-lex.europa.eu/legal-content/en/TXT/?uri=CELEX:32000L0070 accessed December 2018]
14. Council Directive 90/385/EEC of 20 June 1990 on the approximation of the laws of the Member States relating to active implantable medical devices 1990;189(20/07):0017-36.
15. Kramer DB, Xu S, Kesselheim AS. Regulation of medical devices in the United States and European Union: Mass Medical Soc, 2012.
16. European Commission. New EU rules to ensure safety of medical devices. European commission - Fact Sheet, 2017.
17. Legislation.gov.uk. The Human Medicines Regulations 2012 2012 [Available from: http://www.legislation.gov.uk/uksi/2012/1916/regulation/8/made 2018.
18. National Institute for Health and Care Excellence. Interventional procedures guidance 2003 [Available from: https://www.nice.org.uk/about/what-we-do/our-programmes/nice-guidance/nice-interventional-procedures-guidance accessed February 2018]
19. Department of Health. Health Service Circular (HSC2003/011) – The Interventional Procedures Programme 2003 [Available from: https://webarchive.nationalarchives.gov.uk/20120503190439/http://www.dh.gov.uk/prod_consum_dh/groups/dh_digitalassets/@dh/@en/documents/digitalasset/dh_4064925.pdf accessed February 2018]
20. Biffl WL, Spain DA, Reitsma AM, et al. Responsible development and application of surgical innovations: a position statement of the Society of University Surgeons. 2008;206(6):1204-09.
21. Stefanidis D, Fanelli RD, Price R, et al. SAGES guidelines for the introduction of new technology and techniques. *Surg Endosc* 2014;28(8):2257-71. doi: 10.1007/s00464-014-3587-6 [published Online First: 2014/06/19]
22. Royal College of Surgeons. Good Surgical Practice - Introduction of new techniques 2014 [Available from: https://www.rcseng.ac.uk/standards-and-research/gsp/domain-1/1-2-4-introduction-of-new-techniques/ accessed February 2018]
23. Thavaneswaran P, Maddern G, Spigelman A, et al. A review of policies and processes for the introduction of new interventional procedures. ASERNIP-S Report No. 58. Adelaide, South Australia: ASERNIP, 2007.23.

For peer review only - http://bmjopen.bmj.com/site/about/guidelines.xhtml

24. Freedman DB, Swanson E. Introducing new interventional procedures within a District General Hospital, linking with the process for implementing NICE guidance. 2006;11(3):180-86. doi: doi:10.1108/14777270610683100
25. Spigelman AD. Governance and innovation: experience with a policy on the introduction of new interventional procedures. *ANZ J Surg* 2006;76(1-2):9-13. doi: 10.1111/j.1445-2197.2006.03648.x [published Online First: 2006/02/18]
26. Danjoux NM, Martin DK, Lehoux PN, et al. Adoption of an innovation to repair aortic aneurysms at a Canadian hospital: a qualitative case study and evaluation. *BMC Health Serv Res* 2007;7:182. doi: 10.1186/1472-6963-7-182 [published Online First: 2007/11/17]
27. Sharma B, Danjoux NM, Harnish JL, et al. How are decisions to introduce new surgical technologies made? Advanced laparoscopic surgery at a Canadian community hospital: A qualitative case study and evaluation. *Surg Innov* 2006;13(4):250-6. doi: 10.1177/1553350606296341 [published Online First: 2007/01/18]
28. NHS Digital. Estates Return Information Collection, England, 2016-2017 2017 [Available from: https://digital.nhs.uk/data-and-information/publications/statistical/estates-returns-information-collection/estates-return-information-collection-2016-17 accessed February 2018]
29. JISC. Online Surveys 2017 [Available from: https://www.onlinesurveys.ac.uk accessed 12/02/2018.
30. Bryman A. Social research methods: Oxford university press 2016.
31. Braun V, Clarke, V., & Terry, G. . Thematic Analysis. In: Rohleder PL, A., ed. Qualitative research in clinical and health psychology. Basingstoke: Palgrave Macmillan 2014.
32. Health Research Authority. HRA Defining Research 2017 [Available from: http://www.hra-decisiontools.org.uk/research/docs/DefiningResearchTable_Oct2017-1.pdf accessed February 2018]
33. Gornall J. The trial that launched millions of mesh implant procedures: did money compromise the outcome? 2018;363:k4155. doi: 10.1136/bmj.k4155 %J BMJ

Figure legends

Figure 1. Approach to trusts and health boards and planned data extraction from policies for the introduction of new invasive procedures and devices

For peer review only - http://bmjopen.bmj.com/site/about/guidelines.xhtml

Figure 1. Approach to trusts and health boards and planned data extraction from policies for the introduction of new invasive procedures and devices.

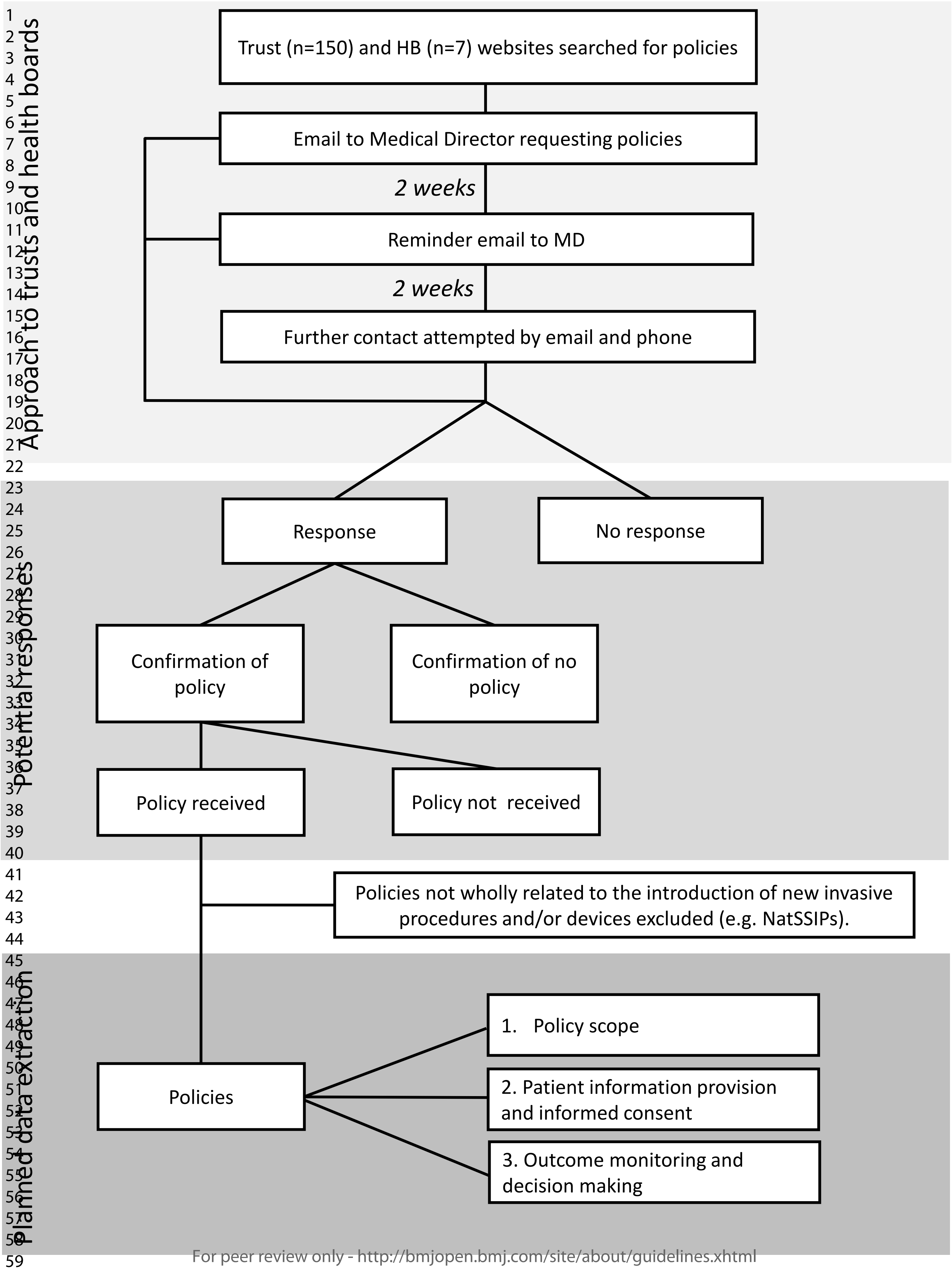

For peer review only - http://bmjopen.bmj.com/site/about/guidelines.xhtml

HB = Health Board

Supplementary file 1. Figure showing the relationship between NICE and the NHS, including roles and structure

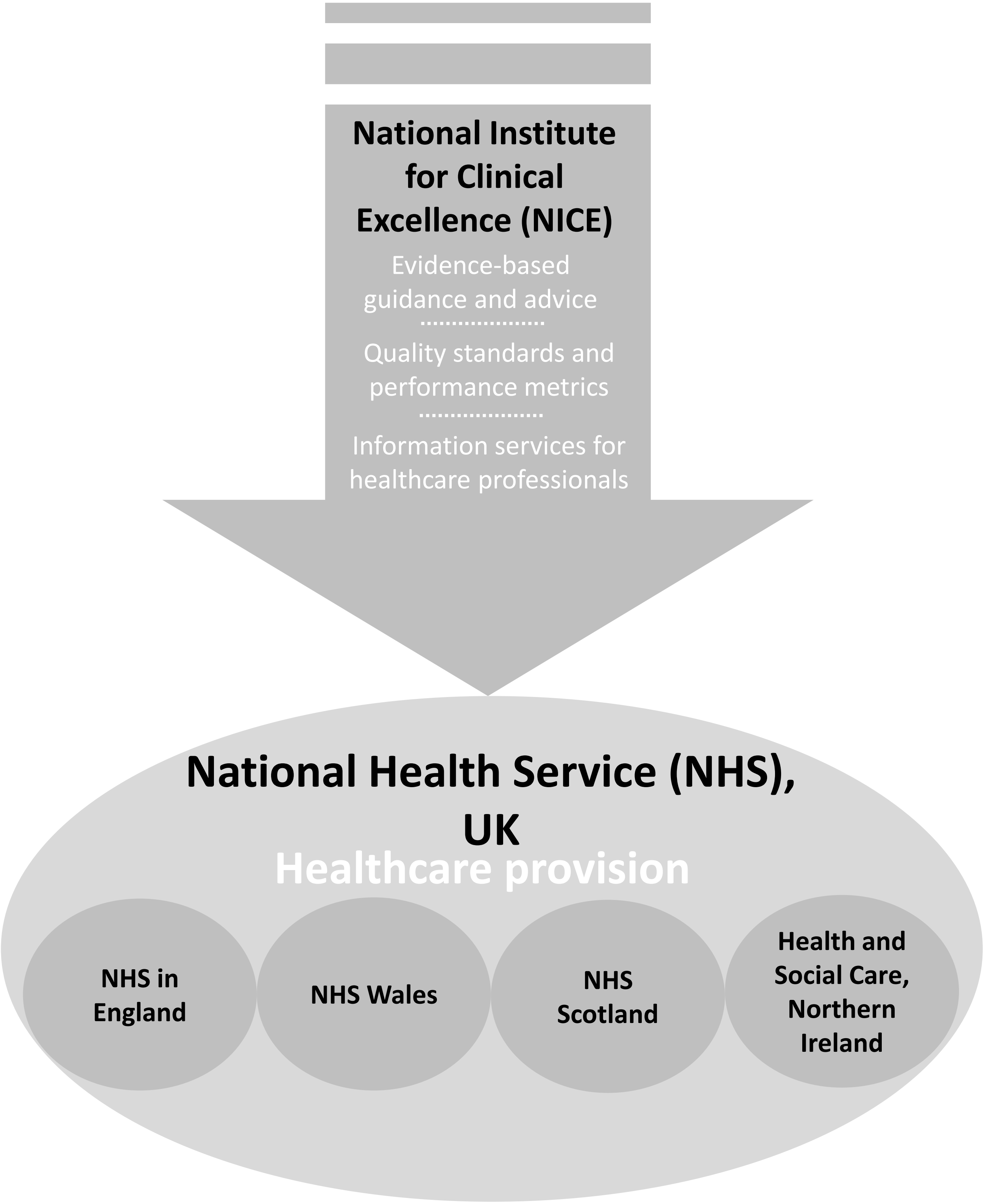

Supplementary file 2. Preliminary data extraction form

**DATA EXTRACTION DETAILS**			
**1**	**Who is extracting the data? (initials)**		
	[ ][ ][ ]		
**2**	**What is the name of the trust?**		
**3**	**What is the Trust ID?**		
	[ ][ ][ ]		
**4**	**Policy title:**		
**5**	**Current policy and/or version number:**		
	[ ][ ][ ]		
**SCOPE OF POLICY**			
**1**	**What procedures/devices does the policy apply to?**		
**a)**	**Invasive procedures**		
	☐ Yes	☐ No	☐ N/A
	If yes, answer i) - ii). If no or not stated go to question b).		
	**i) What term is used to describe these procedures?**		
	**ii) Does the policy define invasive procedures (or similar term used)?**		
	☐ Yes	☐ No	
	If yes -		
	• Copy and paste definition:		
**b)**	**Invasive devices**		
	☐ Yes	☐ No	☐ N/A
	If yes answer i)-iv). If no or not stated go to c).		
	**i) What term is used to describe invasive devices?**		
	**ii) Does the policy define invasive devices (or similar term used)?**		
	☐ Yes	☐ No	
	If yes -		
	• Copy and paste definition:		

2	**When should the policy be implemented?**		
a)	**When procedures/devices are new (or a similar term that implies this)?**		
	☐ Yes (explicitly states that the policy should be implemented in this instance)		
	☐ No (explicitly states that the policy should not be implemented in this instance)		
	☐ Unclear		
	☐ Not stated (does not state whether or not policy should be implemented in this instance)		
	If yes, no or unclear answer i)-iii). If not stated go to b).		
	**i) Does the policy define 'new' (or similar term)?**		
	☐ Yes	☐ No	
b)	**When procedures/devices are modified (to include references to 'changes in clinical practice') (or a similar term that implies this)?**		
	☐ Yes (explicitly states that the policy should be implemented in this instance)		
	☐ No (explicitly states that the policy should not be implemented in this instance)		
	☐ Unclear		
	☐ Not stated (does not state whether or not policy should be implemented in this instance)		
	If yes, no or unclear answer i)-iii). If not stated go to c).		
	**i) Does the policy define 'modification' (or similar term)?**		
	☐ Yes	☐ No	
c)	**When a procedure/device is used within a research study**		
	☐ Yes (explicitly states that the policy should be implemented in this instance)		
	☐ No (explicitly states that the policy should not be implemented in this instance)		
	☐ Unclear		
	☐ Not stated (does not state whether or not policy should be implemented in this instance)		
	i) Copy and paste all text: (if none, type N/A) [QUALITATIVE DATA]		
d)	**When procedures/devices approved by research ethics are used outside of the specified protocol**		
	☐ Yes (explicitly states that the policy should be implemented in this instance)		
	☐ No (explicitly states that the policy should not be implemented in this instance)		

	☐ Unclear		
	☐ Not stated (does not state whether or not policy should be implemented in this instance)		
	i) Copy and paste all text: (if none, type N/A) [QUALITATIVE DATA]		
**e)**	**Other. Please state any other instances in which the policy should be implemented (if none, type N/A):**		
**PATIENT INFORMATION AND CONSENT**			
**1**	**Does the policy state that patients should receive information and/or complete a consent form that is specific to the new procedure/device delivered?**		
	☐ Yes	☐ No	☐ Unclear
	If yes or unclear answer a)-c). If no go to section I.		
**a)**	**Is the applicant required to submit the patient information leaflet and/or consent form with the application?**		
	☐ Yes	☐ No	☐ Unclear
**b)**	**Does the policy provide guidance on what details should be included in the patient information leaflet and/or consent form?**		
	☐ Yes	☐ No	☐ Unclear
	If yes, answer i). If no or unclear go to section I.		
	**i) Does this include informing patients that the procedure/device is new/modified?**		
	☐ Yes	☐ No	☐ Unclear
**c)**	**Does the policy state that specific PIL information should be provided for all approved procedures/devices?**		
	Yes - specific PIL information should be provided for all new procedures/devices.		
	No - specific PIL information is only required in a subset of new procedures/devices (i.e. if there is no NICE Guidance in place).		
	Not stated		
**AUDIT AND OUTCOME MONITORING**			
**1**	**Does the policy state that the use of new procedures/devices should be monitored/reviewed in any way?**		
	☐ Yes	☐ No	☐ N/A
	If yes, go to question 2. If no or not stated go to section H.		
**2**	**Does the policy require applicants to submit outcome data, including adverse events, clinical complications etc?**		

	☐ Yes	☐ No	☐ Unclear
	If yes, answer a)-d). If no or unclear go to question 4.		
**a)**	**Who is responsible for submitting monitoring/outcome data? (tick all that apply)**		
	☐ Individual clinician		
	☐ Head of department		
	☐ Other. Please state:		
	☐ Not stated		
**b)**	**Who is the monitoring/outcome data submitted too? (tick all that apply)**		
	☐ Trust committee responsible for the introduction of new procedures/devices		
	☐ NICE		
	☐ Audit & Effectiveness Dept/Group		
	☐ Clinical effectiveness/safety group		
	☐ Clinical audit group		
	☐ Medical and/or Nursing Director		
	☐ Clinical governance group		
	☐ Other. Please state:		
	☐ Not stated		
**c)**	**Does the policy specify the type of monitoring/outcome data to be submitted?**		
	☐ Yes	☐ No	
	If yes, answer i)-ii). If no go to question d).		
	i) Which of the following outcomes are specified? (tick all that apply)		
	☐ Adverse events		
	☐ Safety		
	☐ Effectiveness		
	☐ Cost related outcomes		
	☐ Near misses		
	☐ Patient Experience/PROMS		
	☐ Other. Please state:		
**3**	**When does the policy state that the procedure should be reviewed/outcome data submitted?**		
	☐ After a certain length of time. *If selected answer a).*		
	☐ After a certain number of cases. *If selected answer b).*		
	☐ This is decided on a case by case basis or according to level of approval/recommendation given by the committee/group.		
	☐ Unclear. *If selected answer c)*		
**a)**	**How many months?**		
	[ ][ ][ ][ ]		
	Go to question 6		
**b)**	**How many cases?**		

	[ ][ ][ ][ ]		
	Go to question 6		
**c)**	**Copy and paste text:**		
**4**	**Does the policy outline when the procedure/device may be used in clinical practice without continuing review via this policy? (ie. when the procedure/device can be adopted in to routine clinical practice)**		
	☐ Yes	☐ No	☐ Unclear
	If yes or unclear, answer a). If no go to question 8.		
**a)**	**Copy and paste text:**		
**5**	**Does the policy outline when or why the use of a new procedure may be stopped/abandoned/immediately reported to MD?**		
	☐ Yes	☐ No	☐ Unclear
	If yes or unclear, answer a). If no go to section H.		
**a)**	**Copy and paste text:**		
**ADDITIONAL NOTES**			
**1**	**Please record any additional comments here. These may relate to data extraction or may be reflections on the policy.**		

For peer review only

Supplementary file 3. Example preliminary topic guide for interviews with clinicians

- Introductory remarks, including taking verbal consent

- Can you tell me about yourself?
  - What is your current position and role?
  - Can you describe the range /type(s) of surgical procedures you undertake?
  - Particular areas of interest/expertise?

- As you know, we are looking at innovation in surgery. Can you talk me through how you would define innovation? (Newness; degree of change, level of risk, impact?)

- Have you experienced an innovative procedure being implemented into practice?
  - If so, can you describe it to me? What made it innovative?
  - What about a time when you have had to learn a new procedure?
  - Potential Prompts: How/where implemented? How did you learn about it? Any changes/refinements over time?
  - Was the procedure monitored in anyway?
  - What has happened to it now?

- If you or a colleague did develop an innovative procedure, do you think ethics approval is needed?
- Is there a procedure for implementing an innovative procedure at your hospital?
- Do you think patients should be informed about the new procedure? What aspects important to communicate?
  - How do you think patients will respond to innovative procedures? What does 'informed consent' mean to you?

- Ending the interview: Is there anything else that I haven't mentioned? Anyone else you recommend I could contact?

For peer review only
